# Relationship Between Digital Creativity, Parenting Style, and Adolescent Performance

**DOI:** 10.3389/fpsyg.2019.02487

**Published:** 2019-11-07

**Authors:** María del Carmen Pérez-Fuentes, María del Mar Molero Jurado, Nieves Fátima Oropesa Ruiz, María del Mar Simón Márquez, José Jesús Gázquez Linares

**Affiliations:** ^1^Department of Psychology, University of Almería, Almería, Spain; ^2^Universidad Politécnica y Artística del Paraguay, Asunción, Paraguay; ^3^Department of Psychology, Universidad Autónoma de Chile, Santiago, Chile

**Keywords:** academic performance, adolescence, digital creativity, family-school relationship, parenting style

## Abstract

**Introduction**: Today’s adolescents live immersed in the digital world and are much more familiarized with the use of electronic devices. At the same time, the new technologies have become established as a powerful resource in teaching and learning, providing new texts where the limits of time and space are overcome. Digital creativity is part of people’s daily lives and must be developed from the school and family context.

**Objective**: The objective of this study was to analyze the relationship among digital creativity, parenting style, and academic performance.

**Method**: This analysis was carried out in a sample of 742 adolescents in Middle School and High School aged 13–19. Digital creativity was evaluated using the Creative Behavior Questionnaire: Digital (CBQD). The Parenting Style Scale was used to evaluate the perception teenage boys and girls who have the various dimensions of their parents’ educational style.

**Results:** Parenting styles were established as a mediating variable in the relationship between digital creativity and academic performance.

**Conclusion:** The roles of digital creativity, which is proposed as a facilitating tool in teaching, and parenting styles in academic performance for improving the family-school relationship are discussed.

## Introduction

The new technologies are considered a powerful resource in teaching and learning because they are providing new contexts where the limits of time and space are overcome. They facilitate collaboration, innovation, and creativity in individuals and organizations (Ala-Mutka et al., 2008). The quality of teaching today goes through recognition of the wide variety of stimuli and possibilities opened through the use of the new technologies. Youths, although they have beliefs about their level of competence in managing different virtual tools (García-Martín et al., 2014), have more knowledge in the use of social networks (Cabero-Almenara and Díaz, 2014).

### What Is Digital Creativity?

Two different focuses on the study of creativity can be found in the literature. First, the more artistic or scientific side of creativity from the perspective of skills linked to music, dancing, painting, sculpture, literature, mathematics, and physics, where authors such as Amabile (1985), Gardner (1993), and Csikszentmihalyi (1996) and others have made enormous contributions. Second, studies on creativity from another perspective, a more everyday approach to creativity, do not require predominance of scientific skills or artistic abilities and are mainly the one in people’s daily lives. The studies by Richards et al. (1988) and Runco (2004) should be mentioned here. In this approach, everyday creativity has been defined as self-expression in daily activities, interpersonal style, professional activities, and daily problem solving (Richards et al., 1988; Torrance, 1988). Both types of creativity are moderately related to each other, since similar psychological processes are involved in not only both, but also different, as mentioned by Ivcevic (2007). Thus, everyone is considered to have a potential for creativity, which can be manifested in many ways, due partly to the plasticity of our brains (Sun et al., 2016); creative thought having been empirically found to fluctuate at different times, increasing in adolescence (Claxton et al., 2005; Kim, 2011).

At the present time, we are witnessing huge digital transformations in a globalized world where technological changes occur in a practically inappreciable time. The new adolescents grow up immerged in the digital world and are much more familiarized with the use of electronic devices (cell phones, computers, tablets, and consoles). Following this reasoning, digital creativity is part of the daily life of today’s youth and has been defined based on three main components: digital creative achievement, school-based everyday creativity, and self-expressive digital creativity (Hoffmann et al., 2016). Creative achievement actually refers to elements in which a person has achieved something with credibility. School-based everyday creativity usually includes elements of digital creativity, which the students may have done at school or for homework. Finally, self-expressive creativity is other creative efforts, which the students have made but have not usually received the same external validation as the creative achievement component. Thus, anyone can experience digital creativity. This study concentrates on school-based everyday creativity.

Some authors have suggested that the benefits of using the new technologies for teaching and learning depend on the learning approach used, teacher skills and coexistence in support settings for students and faculty (Ala-Mutka et al., 2008; Fernández-Batanero and Rodríguez-Martín, 2017), and being able to avoid maladjusted behavior that has been related to low academic performance (Pérez-Fuentes et al., 2011; Estévez et al., 2018). Concerning the focus on learning, some studies have demonstrated that if methodologies developing academic performance of the student (fluidity, flexibility, originality, and elaboration) are used from early ages, their academic performance improves (Guilford, 1950; Renzulli et al., 1986). Thus, we believe that creativity applied to the digital world can be a resource for teaching-learning that could increase academic performance of high school students. Based on this hypothesis, and keeping in mind that digital creativity is a novel subject, and therefore, little studied at the present time, one of the objectives of this study was to analyze school-based everyday creativity, as defined in the paragraph above, and its relationship with academic performance.

### Dimensional Approach to Parenting Styles in Adolescent Development

Parenting style refers to how parents and their children act, behave, and relate to each other in any everyday situation. Since Baumrind (1968) distinguished among the authoritarian, permissive, and democratic parenting styles, much research has been done on its influence in the psychological adjustment of children and adolescents. Later, Maccoby and Martin (1983) differentiated a fourth parenting style, negligent parenting, and since then, numerous studies have tried to corroborate these findings from different perspectives and approaches.

The difference between dimensional and typological approaches in the study of parenting styles should also be mentioned. The dimensional approach, which is the subject of this study, and according to Oliva et al. (2007), is made up of six dimensions, which can explain the relationships of parents with their adolescent children and of children toward their parents: *affect* and communication (emotional nearness, support, harmony, and cohesion), *promotion of autonomy* (respect for the decisions of the minor through conversations where agreements are tolerated), *behavioral control* (set rules, limits, penalties, responsibilities, supervision, and monitoring of behavior), *psychological control* (lack of respect for individuality, intrusive control, manipulation, induction of guilt, emotional blackmail, or withdrawing affect), *self-disclosure* (spontaneous communication by the adolescent about activities, friends, and partner), and *humor* (relaxed aptitude, cheerful, and optimistic). Oliva et al. (2008) described three parenting styles according to the dimensions above: democratic parents (with high levels in all the dimensions mentioned except psychological control, medium levels in behavioral control), strict (strong behavioral, psychological, and affective control), strict (strong behavioral, psychological, and affective control), and indifferent (strong psychological control and weak in rest of dimensions).

There is quite good agreement among researchers on parenting styles that affect and communication have a primary role in adolescent adjustment (Oliva et al., 2008; Fuentes et al., 2015; Riquelme et al., 2018). Pelegrina et al. (2002) stated that affect and communication by parents are an indispensable condition for achieving adequate behavior in their children, optimum self-esteem and self-confidence. Psychological control, on the other hand, is considered independent of promoting autonomy (Silk et al., 2003), although related (Hauser-Kunz and Grych, 2013), especially when promotion of autonomy is understood as promotion of volitional functioning (Soenens and Vansteenkiste, 2010). Meanwhile, psychological control is associated negatively with psychological adjustment (Barber et al., 2012; Cui et al., 2014), in particular, among adolescents who have difficulties in regulating their emotions (Cui et al., 2014) and need psychological intervention (Ho et al., 2018). Although behavioral control by parents in adolescence usually prevents externalizing problems (Silk et al., 2003; Parra and Oliva, 2006; Ruiz-Hernández et al., 2018), the many possible combinations between the dimensions characterizing parenting styles mentioned above and the sensitivity of parents toward the adolescent’s own characteristics must also be kept in mind. In this direction, Oliva et al. (2008) found that when behavior control combines with self-disclosure, adolescent adjustment is more positive. Furthermore, other studies have found that the most effective monitoring favors spontaneous communication (self-disclosure) by adolescents toward their parents (Stattin and Kerr, 2000; Gracia et al., 2012; Jiménez-Iglesias et al., 2013). Other family functioning variables (among which are affect or fondness of members of the family) have also been associated with fewer externalizing problems (in particular with adolescent aggressive behavior) (Pérez-Fuentes et al., 2019).

### Digital Creativity, Parenting Styles, and Academic Performance

In the review of previous research on the relationship between digital creativity and parenting styles, some studies showed significant positive correlations between a permissive style and creativity (Miller et al., 2012) and negative correlations between the authoritarian parenting style and creativity (Fearon et al., 2013). However, in the study by Jackson et al. (2011), affect and control by parents were not significantly related to the use of the new technologies or their children’s creativity.

Moreover, the relationship between parenting style and academic performance has been extensively studied from a psychoeducational perspective. Here we briefly comment on some of the most relevant results of studies reviewed. One previous study done by Pelegrina et al. (2002), on the typologies of parenting styles (democratic, permissive, authoritarian, and indifferent) and academic performance, with a sample of adolescents aged 11–15, revealed that children who perceived their parents as democratic or permissive had higher scores in academic areas. Later, Hernando et al. (2012) found results in the same direction, but in this case with regard to the different dimensions of parenting styles, such that the dimensions of behavior control and disclosure were significantly positively associated with academic performance, while psychological control and humor were negatively correlated. More recently, Rodríguez et al. (2018) found that the permissive style of both parents had a more positive influence on getting better grades and that both the permissive and democratic styles of father and mother were associated with stronger involvement at school. Other studies on how school and family can improve academic performance with digital methodologies and tools (Pérez-Fuentes et al., 2015) found that they improve academic motivation as a major part of learning and achievement behavior (González, 2018), to increase constructive thinking and psychological wellbeing (Quevedo-Aguado and Benavente, 2018).

Based on these empirical findings, the main objective of this study focused on the relationship between school-based everyday creativity and academic performance and the mediating role of parenting styles in this relationship. In addition to exploring the behavior of these variables in this sense, it also analyzed the predictive value of school-based everyday creativity and parenting styles for adolescent academic performance. In this respect, it was expected that parenting styles would exert a mediating effect on the relationship between school-based everyday creativity and academic performance in adolescence and that school-based everyday creativity and parenting styles would partially explain the academic performance of adolescents.

## Method

### Participants

The participants were selected by random sampling. Inclusion criteria were that the participants must be in high school, and exclusion criteria were not speaking the language well or having learning problems that caused them to be unable to answer the questionnaires on their own. This analysis was carried out in a total sample of 742 adolescents from five public middle schools and high schools (55.8 and 44.2%, respectively), in Almeria with aged 13–19 (*M* = 15.63; SD = 1.24). The sample of adolescents had a similar distribution of boys (46.7%) and girls (53.3%).

### Instruments

Academic performance was measured based on *ad hoc* dichotomous yes/no questions on whether they had ever failed a subject or had ever repeated a year. Some authors use grade average as an optimum measure of academic performance (Lamas, 2015).

#### Everyday School-Based Activity

The Everyday School-Based Activity subscale of the Creative Behavior Questionnaire: Digital (CBQD) designed by Hoffmann et al. (2016) was used. The subscale is comprised of 10 items expressed on a Likert-type scale from 1 (*never*) to 5 (*4 or more times*). It is a self-reported measure of creative behavior at school. The CBQD has adequate reliability and validity (Hoffmann et al., 2016). In our sample for the School-Based Everyday Creativity Subscale, the Cronbach’s *α* was 0.625. Some examples of the items on this scale are: *How many times in the last 3 months … Have you made presentations using PowerPoint, Prezi, KeyNote or others? How many times in the last 3 months … Have you made videos or movies using an app (a video app, for example)? How often in the last year, did you develop a blog or website for a class or a school project?*

#### Parenting Style

The Parenting Style Scale (Álvarez-García et al., 2016) was used. This is an adaptation of the instrument designed by Oliva et al. (2007). It has 24 items referring to the adolescent’s perception of the educational style of their parents grouped in six dimensions: affect and communication, promotion of autonomy, behavioral control, psychological control, self-disclosure, and humor. In this study, the Cronbach’s *α* for each of the subscales was 0.843, 0.814, 0.687, 0.710, 0.800, and 0.817, respectively.

### Procedure

The study was approved by the Bioethics Committee of the University of Almeria (Ref: UALBIO2018/015). In all cases, the ethical standards of research were compiled by using an informed consent sheet, and the ethical principles of the declaration of Helsinki were respected. To acquire the data, the management teams at the schools were contacted, and dates, schedules, and groups the instruments would be applied to were agreed upon.

### Data Analysis

The study is a descriptive and correlational cross-sectional design. First, the correlation analyses were performed to explore the relationship between the variables, and the descriptive statistics were presented. For comparison of the fail/no fail subject and repeat/no repeat year groups, a Student’s *t* test was performed with the Cohen’s *d* for effect size estimation.

Then binary logistic regression models were estimated using the enter method. For this, the dependent variables in each case were Fail subject and Repeat year, with the dichotomous answer (yes/no). The predictor variables included were digital creativity (i.e., the School-Based Everyday Creativity Subscale) and parenting styles (Affect and communication, Promotion of autonomy, Behavior control, Psychological control, Self-disclosure, and Humor). The SPSS Statistical Package ver. 23.0 for Windows was used for data processing and analysis.

Finally, to perform the simple mediation analysis, the predictor variable was, in each case, having failed a subject or not and having repeated a year or not, respectively. In each case, as possible mediators, parenting styles that had resulted in involving the logistic equation were entered. For computation of the mediation models, the PROCESS macro for SSPS (Hayes, 2013) was used, applying bootstrapping with coefficients estimated from 5,000 bootstraps.

## Results

### Digital Creativity and Parenting Style: Correlations and Descriptive Statistics

As shown in Table 1, school-based everyday creativity correlated positively with parenting styles: Affect and communication (*r* = 0.12, *p* < 0.01), Promotion of autonomy (*r* = 0.09, *p* < 0.05), Behavioral control (*r* = 0.10, *p* < 0.01), Self-disclosure (*r* = 0.19, *p* < 0.001), and Humor (*r* = 0.10, *p* < 0.01).

**Table 1:** Digital creativity and parenting styles. Correlations and descriptive statistics (N=742).

	SEC	A_C	P_A	B_C	P_C	S_D	HU
SEC	—						
A_C	0.12^**^	—					
P_A	0.09^*^	0.67^***^	—				
B_C	0.10^**^	0.14^***^	0.02	—			
P_C	−0.04	−0.31^***^	−0.42^***^	0.29^***^	—		
S_D	0.19^***^	0.55^***^	0.46^***^	0.20^***^	−0.17^***^	—	
HU	0.10^**^	0.65^***^	0.57^***^	0.14^***^	−0.28^***^	0.51^***^	—
*M*	2.17	13.06	12.80	12.85	9.20	10.50	12.69
*SD*	0.58	2.82	2.77	2.62	2.92	3.33	2.69

*SEC, School-based everyday creativity; A_C, Affect and communication; P_A, Promotion of autonomy; B_C, Behavioral control; P_C, Psychological control; S_D, Self-disclosure; HU, Humor. Correlations and descriptive statistics (*N* = 742)*.

* *p < 0.05*;

** *p < 0.01*;

*** *p < 0.001*.

Table 2 presents the means in school-based everyday creativity, and each of the parenting styles, when groups who had failed a subject and those who had never failed, was compared. As observed in the table, the students who had failed a subject (*M* = 2.28, SD = 0.53) scored significantly lower in creativity (*t* = −3.36, *p* < 0.01; *d* = 0.28) than the group who had not failed (*M* = 2.12, SD = 0.58). The differences between the two groups with regard to parenting styles were in favor of those who had never failed a subject, scoring significantly higher in Affect and communication (*t* = −4.27, *p* < 0.001, *d* = 0.35), Promotion of autonomy (*t* = −3.52, *p* < 0.001, *d* = 0.29), Behavioral control (*t* = −3.25, *p* < 0.01, *d* = 0.27), Self-disclosure (*t* = −5.14, *p* < 0.001, *d* = 0.42), and Humor (*t* = −2.41, *p* < 0.05, *d* = 0.20). The Psychological control style had significantly higher scores (*t* = 3.92, *p* < 0.001, *d* = 0.32) in the group who had suspended a subject (*M* = 9.45, SD = 3.08) than those who had not (*M* = 8.58, SD = 2.45).

**Table 2:** Digital creativity and parenting style. Descriptive statistics and *t* test by failed subject/repeated year.

	Failed subject						*t*	*p*
	Yes			No				
	*N*	Mean	SD	*N*	Mean	SD		
School-based everyday creativity	481	2.12	0.58	215	2.28	0.53	−3.36^**^	0.001
Affect and communication	481	12.78	2.90	215	13.71	2.51	−4.27^***^	0.000
Promotion of autonomy	481	12.57	2.81	215	13.35	2.58	−3.52^***^	0.000
Behavioral control	481	12.64	2.67	215	13.32	2.45	−3.25^**^	0.001
Psychological control	481	9.45	3.08	215	8.58	2.45	3.92^***^	0.000
Self-disclosure	481	10.06	3.30	215	11.45	3.22	−5.14^***^	0.000
Humor	481	12.52	2.76	215	13.03	2.48	−2.41^*^	0.016
	**Repeated year**						***t***	***p***
	**Yes**			**No**				
	***N***	**Mean**	**SD**	***N***	**Mean**	**SD**		
School-based everyday creativity	149	2.01	0.58	552	2.22	0.56	−3.93^***^	0.000
Affect and communication	149	12.24	3.25	552	13.28	2.65	−3.48^**^	0.001
Promotion of autonomy	149	12.36	2.80	552	12.92	2.74	−2.15^*^	0.031
Behavioral control	149	12.25	2.83	552	13.01	2.55	−2.86^**^	0.005
Psychological control	149	9.69	3.21	552	9.06	2.84	2.10^*^	0.036
Self-disclosure	149	9.79	3.23	552	10.68	3.34	−2.84^**^	0.005
Humor	149	12.48	3.14	552	12.74	2.55	−0.94	0.346

*Descriptive statistics and *t* test by failed subject/repeated year*.

The comparison of repeaters/not repeaters with respect to school-based everyday activity, as observed in the table, showed that repeaters (*M* = 2.01, SD = 0.58) scored significantly lower (*t* = −3.93, *p* < 0.001, *d* = 0.36) than the group of non-repeaters (*M* = 2.22, SD = 0.56). In addition, by parenting styles, significant statistical differences were observed in which the repeaters (*M* = 9.69, SD = 3.21) had higher scores in the Psychological control style (*t* = 2.10, *p* < 0.05, *d* = 0.19) than non-repeaters (*M* = 9.06, SD = 2.84), whereas non-repeaters had higher mean scores in the Affect and communication (*t* = −3.48, *p* < 0.01, *d* = 0.32), Promotion of autonomy (*t* = −2.15, *p* < 0.05, *d* = 0.20), Behavioral control (*t* = −2.86, *p* < 0.01, *d* = 0.26), and Self-disclosure (*t* = −2.84, *p* < 0.01, *d* = 0.26) styles.

### Logistic Regression Model: Fail a Subject

The dependent variable for logistic regression was having failed a subject or not. The predictor variables entered in the equation were school-based everyday creativity and parenting styles (Affect and communication, Promotion of autonomy, Psychological control, Self-disclosure, and Humor). These variables, the regression coefficients, standard error of estimation, Wald statistic, degrees of freedom, and associated probability, the partial correlation coefficient, and the cross-product ratio are shown in Table 3.

**Table 3:** Results derived from the logistic regression for the probability of failing a subject, by school-based everyday creativity and parenting style.

Variables	*β*	SE	Wald	df	Sig.	Exp (*β*)	95% CI
School-based everyday creativity	−0.322	0.161	3.992	1	0.046	0.725	0.528–0.994
Affect and communication	−0.087	0.053	2.673	1	0.102	0.917	0.826–1.017
Promotion of autonomy	−0.001	0.050	0.000	1	0.986	0.999	0.906–1.102
Behavioral control	−0.120	0.042	8.288	1	0.004	0.887	0.818–0.962
Psychological control	0.113	0.039	8.387	1	0.004	1.119	1.037–1.208
Self-disclosure	−0.081	0.035	5.392	1	0.020	0.922	0.861–0.987
Humor	0.101	0.049	4.152	1	0.042	1.106	1.004–1.218
Constant	2.765	0.881	9.839	1	0.002	15.871	

The odds ratio or cross-product ratio found for each variable shows that (1) the risk of failing a subject is higher in adolescents whose parents’ educational styles are based on psychological control and humor and (2) school-based everyday creativity and behavior control and self-disclosure parenting styles are protective factors against the probability of failing a subject.

The overall fit of the model was (*χ^2^* = 45.68; df = 7; *p* < 0.001) confirmed by the Hosmer-Lemeshow test (*χ^2^* = 7.77; df = 8; *p* = 0.455). The Nagelkerke *R^2^* showed that 10.5% of the variability in the response variable was explained by the logistic regression model. Based on the classification table, a probability of the logistic function being correct was 68.3%, with a false positive rate of 0.843 and a false negative rate of 0.92.

### Logistic Regression Model: Repeat Year

In this case, to perform the logistic regression, the dependent variable was having repeated a year or not, while the predictor variables entered in the equation were, as in the previous model, school-based everyday creativity and the parenting styles. It may be observed in Table 4 that the odds ratio found for each variable revealed that (1) adolescents who scored higher in school-based everyday creativity and whose parents/guardians had a parenting style based on affect and communication have a lower risk of repeating a year, or in other words, these two variables would be acting as protective factors against the probability of repeating and (2) concerning risk factors, control psychological as a parenting style would be significantly involved in the logistic equation.

**Table 4:** Results derived from the logistic regression for the probability of repeat year, according to everyday creativity and parental styles.

Variables	*β*	SE	Wald	df	Sig.	Exp (*β*)	95% CI
School-based everyday creativity	−0.546	0.198	7.590	1	0.006	0.579	0.393–0.854
Affect and communication	−0.166	0.057	8.303	1	0.004	0.847	0.757–0.948
Promotion of autonomy	0.061	0.055	1.224	1	0.269	1.063	0.954–1.185
Behavioral control	−0.078	0.045	3.080	1	0.079	0.925	0.847–1.009
Psychological control	0.091	0.044	4.345	1	0.037	1.095	1.005–1.193
Self-disclosure	−0.010	0.040	0.063	1	0.801	0.990	0.915–1.071
Humor	0.109	0.057	3.628	1	0.057	1.115	0.997–1.247
Constant	−0.073	0.944	0.006	1	0.939	0.930	

Overall fit (*χ^2^* = 29.20; df = 7; *p* < 0.001) was confirmed by the Hosmer-Lemeshow test (*χ^2^* = 11.12; df = 8; *p* = 0.195), while the Nagelkerke *R^2^* coefficient showed that 7.8% of the variance was explained by the logistic regression model. Based on the classification table, probability of the logistic function being correct was 80.7%, with a false positive rate of 0.004 and a false negative rate of 0.

### Mediation Models

Based on these results, we felt the need to find out whether certain parenting styles could be mediating in the relationship between failing a subject/repeating a year and the level of school-based everyday creativity. Therefore, simple mediation models were computed, including the parenting styles involved in the corresponding logistic equation as mediators in each case.

**Figure 1:**
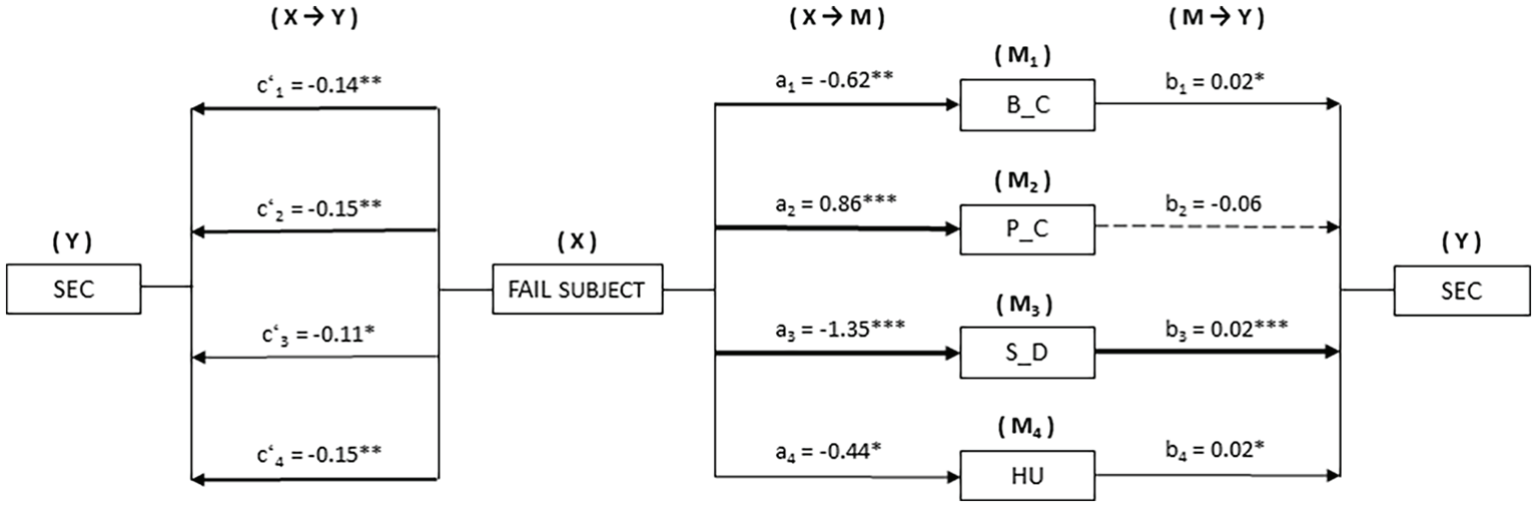
Mediation model for parenting styles on the relationship between failing a subject and school-based everyday creativity. *p < 0.05; **p < 0.01; ***p < 0.001.

Figure 1 shows the mediation models taking fail a subject or not as the independent variable (X). In this case, the behavioral control (M_1_), psychological control (M_2_), self-disclosure (M_3_), and humor (M_4_) parenting styles were entered as possible mediators on the effect in school-based everyday creativity (Y).

In the first place, a significant relationship was observed between failing a subject (X) and parenting styles (M): B_C [*B* = −0.62, *p* < 0.01], P_C [*B* = 0.86, *p* < 0.001], S_D [*B* = −1.35, *p* < 0.001], and HU [*B* = −0.44, *p* < 0.05]. Estimation of direct effects X → Y showed significance of failing a subject on school-based everyday creativity (Y) in each of the models computed: B_C [*B* = −0.14, *p* < 0.01], P_C [*B* = −0.15, *p* < 0.01], S_D [*B* = −0.11, *p* < 0.05], and HU [*B* = −0.15, *p* < 0.01]. Furthermore, estimation of M → Y found significant effects on school-based everyday creativity (Y) in three parenting styles (M): B_C [*B* = 0.02, *p* < 0.05], S_D [*B* = 0.02, *p* < 0.001], and HU [*B* = 0.02, *p* < 0.05].

With the analysis of indirect effects (X → M → Y) with bootstrapping, significance was found in three of four models computed: B_C [*B* = −0.01, SE = 0.007, 95% CI (−0.031, −0.002)], S_D [*B* = −0.04, SE = 0.012, 95% CI (−0.069, −0.018)], and HU [B = −0.009, SE = 0.006, 95% CI (−0.027, −0.000)].

**Figure 2:**
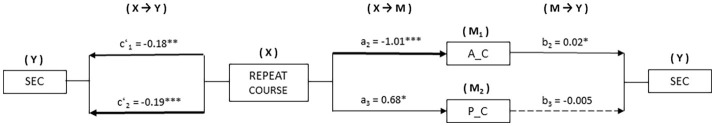
Mediation model of parenting styles on the relationship between repeating a year and school-based everyday creativity. *p < 0.05; **p < 0.01; ***p < 0.001.

Figure 2 presents the mediation models with repeat year or not (X) as the independent variable. In this case, the Affect and communication (M_1_) and Psychological control (M_2_) parenting styles were entered as possible mediators on the effect in school-based everyday creativity (Y).

In the first place, significant relationships were observed between repeating a year (X) and parenting styles (M): A_C [*B* = −1.01, *p* < 0.001] and P_C [*B* = 0.68, *p* < 0.05]. The estimation results of the direct effects (X → Y) revealed the significant relationships of repeating the year (X) on school-based everyday creativity (Y), in computation of both models: A_C [*B* = −0.18, *p* < 0.01] and P_C [*B* = −0.19, *p* < 0.001]. The estimation of the M → Y effects found a significant effect of the A_C parenting style [*B* = 0.02, *p* < 0.05] on school-based everyday creativity.

Finally, with the analysis of the indirect effects (X → M → Y) with bootstrapping, significance was found in the model that took the A_C [*B* = −0.02, SE = 0.010, 95% CI (−0.047, −0.003)] parenting style as the mediator.

## Discussion

Based on the pioneering study by Hoffmann et al. (2016) on digital creativity and its conceptualization in the school, in this study we analyzed the relationship among school-based everyday creativity, parenting styles, and academic performance. Our first conclusion is that parenting styles have a relevant role in school-based everyday creativity and academic performance. These main results are discussed below.

First, with respect to the relationship between *parenting style and digital creativity*, our results showed that adolescents who perceived that their parents had a more democratic relational style, although with higher scores in behavior control, had better results in school-based everyday creativity. In this sense, other researchers, analyzing creativity from a perspective other than digital, have also found significant correlations between young people’s perception of high affect in parenting style and the perception of possessing personal characteristics and creative thinking styles (Miller et al., 2012), pointing in the same direction as our data. In another direction, a study by Fearon et al. (2013) with Jamaican students and their parents found the high level of control, which characterized the authoritarian parenting style to be associated with lower levels of creativity. On the other hand, in the study by Jackson et al. (2011), parental behavior in the dimensions of affect and control (measured through the perception of parents and adolescents) did not significantly correlate with the use of new technologies, or with their child’s creativity, which was measured using the Torrance Creative Thinking Test. With respect to the dimension of parental control, these results could be due to the lack of differentiation by the researchers between the dimensions of behavioral and psychological control in measuring parenting styles, so that in other studies, psychological control, which has been associated with adolescent adjustment problems (Parra and Oliva, 2006; Barber et al., 2012), would have been measured instead of behavioral control. Second, concerning the comparison of *academic performance and digital creativity*, our data showed that those students who did not fail a subject or who had not repeated a year scored higher on school-based everyday creativity. Other researchers have reported similar findings, emphasizing the positive role of the use of creative methodologies as facilitators of more significant student learning (Guilford, 1950; Renzulli et al., 1986; Limiñana et al., 2010). Third, with regard to the *parenting style and academic performance*, we found that the perception of psychological control by adolescents was a risk factor, for both failing a subject and repeating a year. Furthermore, perceived humor in the figure of the parents also contributed to failing a subject, and along with the perception of psychological control may allow ways of parental relating to be seen that do not respect the individualism of the child, forms of relating that have also been associated with low self-esteem (Silk et al., 2003). Other studies have reported similar results (Hernando et al., 2012). Apart from that, in the study by Pelegrina et al. (2002), the perception of democratic and permissive styles by adolescents correlated positively with academic performance, but the perception of parental control did not relate negatively with academic performance, perhaps again because behavioral and psychological control were not differentiated. The results of the study by Rodríguez et al. (2018) support those results.

Fourth, the objective of developing an explanatory model for academic performance based on variables such as digital creativity and parenting styles was met in a logistic regression analysis. The results of that analysis reflected school-based everyday creativity as a factor that can promote adequate academic performance, with other parental variables relevant for not failing a subject, such as the behavioral control and self-disclosure parenting style dimensions, and the affect and communication parenting style dimension for not repeating the year. The model explained 10.5 and 7.8% of adolescent academic performance, respectively. Therefore, both school-based everyday creativity and behavioral control and self-disclosure, on one hand, and the affect and communication parenting style, on the other, contributed to explaining part of adolescents’ academic performance. These variables could probably have explained an even larger proportion of academic performance if the teachers had employed more active teaching learning methodologies. However, most of the schools still use a traditional teaching-learning methodology involving psychological processes, which do not promote creativity, but, on the contrary, limit its development. Other studies have emphasized the preponderant role of creative digital methodologies as a significant facilitating tool for classroom teaching and learning, leading to improved digital competence from an innovative perspective (Guilford, 1950; Renzulli et al., 1986; Ala-Mutka et al., 2008; Limiñana et al., 2010; Fernández-Batanero and Rodríguez-Martín, 2017), making use this way of the high creative potential of the adolescent, which is greater than in other stages of development (Claxton et al., 2005; Kim, 2011).

Fifth, the new analyses performed in this study on the mediating role of parenting style in the relationship between school-based everyday creativity and academic performance revealed that the association between school-based everyday creativity and failing a subject was mediated by the perception of parenting styles (high behavioral control, high self-disclosure, and low psychological control). Furthermore, the relationship between school-based everyday creativity and repeating a year was mediated by the perception of parenting styles with high doses of communication and affect and low psychological control in parent-child interaction.

Among the limitations of this study is its cross-sectional design, which could be completed with longitudinal studies contributing to generalization of the results. Another possible limitation has to do with the fact that the parenting styles were evaluated based only on the perceptions of the adolescents, and other contributions could be added, such as those of teachers and parents. Based on all of the above, more studies are required to enlarge these first results on digital creativity in the school and that include new variables referring to the adolescent, the family, and teachers.

## Conclusions

In view of the above, the results of this study enable two broad conclusions to be arrived at for a possible intervention proposal. It was concluded that parenting styles have a relevant role in developing digital creativity in the school and its relationship with academic performance in adolescence. Therefore, the data suggest that the family continues to play a relevant role as an educating agent in this stage of development, and above all, the parents’ educational style. Thus, the perception of adolescents of a parenting style characterized by high affect and communication, high behavioral control, low psychological control, and high self-disclosure predicts better academic performance in adolescence. This result is important from the point of view of educational intervention to the extent that it demonstrates that intervention with families must be a priority objective of secondary education teachers. Thus, the school could take action with the family in participative, individualized counseling sessions, where teachers can advise the families based on their real needs or interests, with simple, plausible educational patterns contributing to the adolescent’s positive development. Educational projects could also be carried out that include parenting workshops and activities, thereby favoring and improving the family-school relationship.

Considering that the more traditional teaching methodologies and evaluation systems cannot faithfully include adolescent digital creativity, it is concluded that digital creative methodologies must be developed as a significant facilitating tool of classroom teaching and learning for the main goal of improving adolescent academic performance, by applying digital creativity to the school environment. This type of methodology could also be widened to the family environment.

As a future line of research, the educational implications of digital creativity in adolescent development could continue to be studied, including new variables in the design, such as self-concept or self-esteem, agreeableness, and self-efficacy, and in any case, involved directly with personal development. The study of digital creativity could also be widened by adapting the instrument to other contexts and populations.

## Data Availability Statement

The datasets generated for this study are available on request to the corresponding author.

## Ethics Statement

The studies involving human participants were reviewed and approved by the Bioethics Committee of the University of Almeria. Written informed consent to participate in this study was provided by the participants and their legal guardian/next of kin where appropriate. The study was approved by the Bioethics Committee of the University of Almeria (Ref: UALBIO2018/015).

## Author Contributions

MP-F, MM, NO, and MS contributed to the conception and design of the review. JG applied the search strategy. All authors applied the selection criteria, completed the assessment of risk of bias, and analyzed the data and interpreted data. MM, MP-F, MS, and NO wrote this manuscript. MP-F and JG edited this manuscript. MP-F is responsible for the overall project.

### Conflict of Interest

The authors declare that the research was conducted in the absence of any commercial or financial relationships that could be construed as a potential conflict of interest.

## References

[ref1] Ala-Mutka K. Punie Y. Redecker C. (2008). ICT for learning, innovation and creativity. Luxembourg: Institute for Prospective Technological Studies (IPTS), European Commission, Joint Research Center.

[ref2] Álvarez-García D. García T. Barreiro-Collazo A. Dobarro A. Antúnez Á. (2016). Parenting style dimensions as predictors of adolescent antisocial behavior. Front. Psychol. 7:1383. 10.3389/fpsyg.2016.01383 27679591 PMC5020069

[ref3] Amabile T. M. (1985). Motivation and creativity: effects of motivational orientation creative writers. J. Pers. Soc. Psychol. 48, 393–399.

[ref4] Barber B. K. Xia M. Olsen J. A. McNeely C. A. Bose K. (2012). Feeling disrespected by parents: refining the measurement and understanding of psychological control. J. Adolesc. 35, 273–287. 10.1016/j.adolescence.2011.10.010 22177194

[ref5] Baumrind D. (1968). Authoritarian vs. authoritative parental control. Adolescence 3, 255–272.

[ref6] Cabero-Almenara J. Díaz V. M. (2014). Posibilidades educativas de las redes sociales y el trabajo en grupo: Percepciones de los alumnos universitarios [educational possibilities of social networks and group work. University students’ perceptions]. Comun.: Rev. Científica Iberoam. Comun. Educ. 42, 165–172. 10.3916/C42-2014-16

[ref7] Claxton A. F. Pannells T. C. Rhoads P. A. (2005). Developmental trends in the creativity of school-age children. Creat. Res. J. 17, 327–335. 10.1207/s15326934crj1704_4

[ref9] Csikszentmihalyi M. (1996). Creativity. Flow and the psychology of discovery and invention. New York: Harper Collins Publishers.

[ref10] Cui L. Morris A. S. Criss M. M. Houltberg B. J. Silk J. S. (2014). Parental psychological control and adolescent adjustment: the role of adolescent emotion regulation. Parenting: Sci. Prac. 14, 47–67. 10.1080/15295192.2014.880018 PMC4104177 25057264

[ref11] Estévez E. Jiménez T. I. Moreno D. (2018). Aggressive behavior in adolescence as a predictor of personal, family, and school adjustment problems. Psicothema 30, 66–73. Available at: http://www.redalyc.org/articulo.oa?id=72754594011 29363473 10.7334/psicothema2016.294

[ref12] Fearon D. D. Copeland D. Saxon T. F. (2013). The relationship between parenting styles and creativity in a sample of Jamaican children. Creat. Res. J. 25, 119–128. 10.1080/10400419.2013.752287

[ref13] Fernández-Batanero J. M. Rodríguez-Martín A. (2017). TIC y diversidad funcional: conocimiento del profesorado [ICT and functional diversity: knowledge of the teaching staff]. Eur. J. Invest. Health Psychol. Educ. 7, 157–175. 10.30552/ejihpe.v7i3.204

[ref14] Fuentes M. C. García F. Gracia E. Alarcón A. (2015). Los estilos parentales de socialización y el ajuste psicológico. Un estudio con adolescentes españoles [Parental styles of socialization and psychological adjustment. A study with Spanish adolescents]. Rev. Psicodidáctica 20, 117–138. 10.1387/RevPsicodidact.10876

[ref15] García-Martín J. García-Sánchez J. N. Álvarez-Fernández M. L. Díez-Caso H. (2014). Efectos en la competencia digital tras la aplicación de un programa de competencias ocupacionales [Effects on digital competence after application of a program of occupational skills]. Eur. J. Educ. Psychol. 7, 73–81. 10.1989/ejep.v7i2.180

[ref16] Gardner H. (1993). Creating minds. New York: Basic Books.

[ref17] González M. L. G. (2018). Incidencia del sexo, número de hermanos y orden de Nacimiento en las metas académicas de estudiantes universitarios [incidence of sex, number of siblings and birth order in the academic goals of university students]. Eur. J. Child Dev., Educ. Psychopathology 6, 57–66. 10.30552/ejpad.v6i1.62

[ref18] Gracia E. Fuentes M. C. Garcia F. Lila M. (2012). Perceived neighborhood violence, parenting styles, and developmental outcomes among Spanish adolescents. J. Community Psychol. 40, 1004–1021. 10.1002/jcop.21512

[ref19] Guilford J. P. (1950). Creativity. Am. Psychol. 5, 444–454. 14771441 10.1037/h0063487

[ref20] Hauser-Kunz J. Grych J. H. (2013). Parental psychological control and autonomy granting: distinctions and associations with child and family functioning. Parenting 13, 77–94. 10.1080/15295192.2012.709147 23418403 PMC3572750

[ref21] Hayes A. F. (2013). Introduction to mediation, moderation, and conditional Process analysis: A regression-based approach. New York, EE.UU: The Guilford Press.

[ref22] Hernando Á. Oliva A. Pertegal M. Á. (2012). Variables familiares y rendimiento académico en la adolescencia [Family variables and academic achievement in adolescence]. Estud. Psicol. 33, 51–65. 10.1174/021093912799803791

[ref23] Ho S. M. Dai D. W. T. Mak C. Liu K. W. K. (2018). Cognitive factors associated with depression and anxiety in adolescents: a two-year longitudinal study. Int. J. Clin. Health Psychol. 18, 227–234. 10.1016/j.ijchp.2018.04.001 30487928 PMC6224862

[ref24] Hoffmann J. Ivcevic Z. Brackett M. (2016). Creativity in the age of technology: measuring the digital creativity of millennials. Creat. Res. J. 28, 149–153. 10.1080/10400419.2016.1162515

[ref25] Ivcevic Z. (2007). Artistic and everyday creativity: an act-frequency approach. J. Creat. Behav. 41, 271–290. 10.1002/j.2162-6057.2007.tb01074.x

[ref26] Jackson L. A. Witt E. A. Fitzgerald H. E. Von Eye A. Zhao Y. (2011). “Parent behavior, children’s technology use and creativity: videogames count but parents don’t” in Proceedings of the 5th WSEAS international conference on Communications and information technology. eds. Mastorakis N. E. Bojkovicc Z. (Wisconsin, USA: World Scientific and Engineering Academy and Society (WSEAS)), 19–24.

[ref27] Jiménez-Iglesias A. Moreno C. García-Moya I. Ramos P. (2013). How can parents obtain knowledge about their adolescent children? Infanc. Aprendizaje 36, 181–197. 10.1174/021037013806196256

[ref28] Kim K. H. (2011). The creativity crisis: the decrease in creative thinking scores on the Torrance tests of creative thinking. Creat. Res. J. 23, 285–295. 10.1080/10400419.2011.627805

[ref29] Lamas H. A. (2015). Sobre el rendimiento escolar [School Performance]. Propós. represent. 3, 313–386. 10.20511/pyr2015.v3n1.74

[ref30] Limiñana R. M. Bordoy M. Juste G. Corbalán J. (2010). Creatividad, aptitudes intelectuales y estilos de respuesta: implicaciones para el rendimiento académico en secundaria [Creativity, intelectual abilities and response styles: Implications for academic performance in the secondary school]. Ann. Psychol. 26, 212–219. Retrieved from: https://revistas.um.es/analesps/article/view/109081

[ref31] Maccoby E. E. Martin J. A. (1983). “Socialization in the context of the family: parent-child interaction” in Handbook of child psychology, Vol. IV: Socialization, personality and social development. 4th Edn. eds. Hetherington E. M. Mussen P. H. (Nueva York: Wiley), 1–101.

[ref32] Miller A. L. Lambert A. D. Speirs Neumeister K. L. (2012). Parenting style, perfectionism, and creativity in high-ability and high-achieving young adults. J. Educ. Gift. 35, 344–365. 10.1177/0162353212459257

[ref33] Oliva A. Parra Á. Arranz E. (2008). Estilos relacionales parentales y ajuste adolescente [Parenting styles and adolescent adjustment]. Infanc. Aprendizaje 31, 93–106. 10.1174/021037008783487093

[ref34] Oliva A. Parra Á. Sánchez-Queija I. López F. (2007). Estilos educativos materno y paterno: evaluación y relación con el ajuste adolescente [maternal and paternal parenting styles: assessment and relationship with adolescent adjustment]. Ann. Psychol. 23, 49–56. Retrieved from: https://www.redalyc.org/articulo.oa?id=167/16723107

[ref35] Parra Á. Oliva A. (2006). Un análisis longitudinal sobre las dimensiones relevantes del estilo parental durante la adolescencia [Relevant dimensions of parenting style during adolescence: A longitudinal study]. Infanc. Aprendizaje 29, 453–470. 10.1174/021037006778849594

[ref36] Pelegrina S. García-Linares M. C. G. Casanova P. F. (2002). Los estilos educativos de los padres y la competencia académica de los adolescentes [Parenting styles and adolescents’ academic performance]. Infanc. Aprendizaje 25, 147–168. 10.1174/021037002317417796

[ref37] Pérez-Fuentes M. D. C. Álvarez-Bermejo J. A. Molero M. D. M. Gázquez J. J. López M. A. L. (2015). Violencia escolar y rendimiento académico (VERA): Aplicación de realidad aumentada [School violence and academic achievement (vera): Augmented reality application]. Eur. J. Invest. Health, Psychol. Educ. 1, 71–84. 10.1989/ejihpe.v1i2.6

[ref38] Pérez-Fuentes M. D. C. Gázquez J. J. Mercader I. Molero M. D. M. García M. D. M. (2011). Rendimiento académico y conductas antisociales y delictivas en alumnos de Educación Secundaria Obligatoria [Academic achievement and antisocial behavior in public secondary education students]. Int. J. Psychol. Psychol. Ther. 11, 401–412. Retrieved from: https://www.ijpsy.com/volumen11/num3/307.html

[ref39] Pérez-Fuentes M. D. C. Molero M. D. M. Barragán A. B. Gázquez J. J. (2019). Family functioning, emotional intelligence, and values: analysis of the relationship with aggressive behavior in adolescents. Int. J. Environ. Res. Public Health 16, pii: 478. 10.3390/ijerph16030478 PMC6388189 30736326

[ref41] Quevedo-Aguado M. P. Benavente M. H. (2018). Análisis de variables de personalidad, bienestar psicológico y pensamiento constructivo en estudiantes de Ciencias de la Salud [analysis of personality variables, psychological well-being and constructive thinking in students of sciences of health]. Eur. J. Health Res. 4, 5–18. 10.30552/ejhr.v4i1.86

[ref42] Renzulli M. J. Gay-Ford B. Smith L. Renzulli J. (1986). New directions in creativity. Connecticut: Creative Learning Pres, Inc.

[ref43] Richards R. Kinney D. K. Benet M. Merzel A. P. C. (1988). Assessing everyday creativity: characteristics of the everyday creativity scales and validation with three large samples. J. Pers. Soc. Psychol. 54, 467–485.

[ref44] Riquelme M. Garcia O. F. Serra E. (2018). Psychosocial maladjustment in adolescence: parental socialization, self-esteem, and substance use. An. Psicol. 34, 536–544. 10.6018/analesps.34.3.315201

[ref45] Rodríguez L. Revuelta M. Sarasa M. Fernández O. (2018). The role of parental socialization styles in school engagement and academic performance. Eur. J. Educ. Psychol. 11, 123–139. 10.30552/ejep.v11i2.226

[ref46] Ruiz-Hernández J. A. Moral-Zafra E. Llor-Esteban B. Jiménez-Barbero J. A. (2018). Influence of parental styles and other psychosocial variables on the development of externalizing behaviors in adolescents: a systematic review. Eur. J. Psychol. Appl. Leg. Context 11, 9–21. 10.5093/ejpalc2018a11

[ref47] Runco M. A. (2004). “Everyone has creative potential” in Creativity: From potential to realization. eds. Sternberg R. J. Grigorenko E. L. Singer J. L. (Washington, DC: American Psychological Association), 21–30.

[ref48] Silk J. S. Morris A. S. Kanaya T. Steinberg L. (2003). Psychological control and autonomy granting: opposite ends of a continuum or distinct constructs? J. Res. Adolesc. 13, 113–128. 10.1111/1532-7795.1301004

[ref49] Soenens B. Vansteenkiste M. (2010). A theoretical upgrade of the concept of parental psychological control: proposing new insights on the basis of self-determination theory. Dev. Rev. 30, 74–99. 10.1016/j.dr.2009.11.001

[ref50] Stattin H. Kerr M. (2000). Parental monitoring: A reinterpretation. Child Dev. 71, 1072–1085. 10.1111/1467-8624.00210 11016567

[ref51] Sun J. Chen Q. Zhang Q. Li Y. Li H. Wei D. (2016). Training your brain to be more creative: brain functional and structural changes induced by divergent thinking training. Hum. Brain Mapp. 37, 3375–3387. 10.1002/hbm.23246 27159407 PMC6867508

[ref52] Torrance E. P. (1988). “The nature of creativity as manifest in its testing” in The nature of creativity: Contemporary psychological perspectives. ed. Sternberg R. J. (New York, NY, US: Cambridge University Press), 43–75.

